# Assessing environmental attributes and effects of climate change on *Sphagnum* peatland distributions in North America using single- and multi-species models

**DOI:** 10.1371/journal.pone.0175978

**Published:** 2017-04-20

**Authors:** Tobi A. Oke, Heather A. Hager

**Affiliations:** Department of Integrative Biology, University of Guelph, Guelph, ON, Canada; Missouri Botanical Garden, UNITED STATES

## Abstract

The fate of Northern peatlands under climate change is important because of their contribution to global carbon (C) storage. Peatlands are maintained via greater plant productivity (especially of *Sphagnum* species) than decomposition, and the processes involved are strongly mediated by climate. Although some studies predict that warming will relax constraints on decomposition, leading to decreased C sequestration, others predict increases in productivity and thus increases in C sequestration. We explored the lack of congruence between these predictions using single-species and integrated species distribution models as proxies for understanding the environmental correlates of North American *Sphagnum* peatland occurrence and how projected changes to the environment might influence these peatlands under climate change. Using Maximum entropy and BIOMOD modelling platforms, we generated single and integrated species distribution models for four common *Sphagnum* species in North America under current climate and a 2050 climate scenario projected by three general circulation models. We evaluated the environmental correlates of the models and explored the disparities in niche breadth, niche overlap, and climate suitability among current and future models. The models consistently show that *Sphagnum* peatland distribution is influenced by the balance between soil moisture deficit and temperature of the driest quarter-year. The models identify the east and west coasts of North America as the core climate space for *Sphagnum* peatland distribution. The models show that, at least in the immediate future, the area of suitable climate for *Sphagnum* peatland could expand. This result suggests that projected warming would be balanced effectively by the anticipated increase in precipitation, which would increase *Sphagnum* productivity.

## Introduction

Northern peatlands are important for global carbon storage, holding ~30% of global soil carbon [1]. Peatlands develop under cool temperatures and high moisture and thus are tightly regulated by climate [2], [3]. Peatlands accumulate carbon when net primary productivity exceeds decomposition, with decomposition limited by both the waterlogged conditions that characterize peatlands [4], [5] and the decay resistance of *Sphagnum* species, which dominate many northern peatlands. This recalcitrant nature of *Sphagnum* tissue is attributed to the inhibitive effects of its litter chemistry on microbial decomposition [6–8]. *Sphagnum* species contribute up to 50% of organic soil carbon in many northern peatlands [4].

Peatlands are categorized as ombrotrophic (bogs) or minerotrophic (fens) based on their hydrological, chemical, and biotic structure. Bogs are fed by atmospheric water sources and are acidic, nutrient poor, and dominated by *Sphagnum* species. Fens are fed by surface water (e.g. rivers and lakes) and are acidic or alkaline and composed of a mixture of mosses (e.g. *Sphagnum* and feather mosses) and vascular plants (e.g. sedges). Fens are further characterized as rich or poor. Poor fens are acidic, dominated by *Sphagnum*, and are considered one of the successional stages of bog development [9–11]. Globally, *Sphagnum* remains the only ecologically dominant moss group and stores significant amounts of carbon annually [12], [13].

In nature, *Sphagnum* species are distributed along a microtopographical moisture gradient whereby some species typically occur in wetter hollows while others occur on drier hummocks [10], [14], [15]. The water economy of a *Sphagnum* species, and thus its position along the hummock-hollow gradient, is mediated by its growth form. Hummock species maintain tighter stems and dense canopy integration that help prevent desiccation [16–18], whereas hollow species tend to maintain a loose canopy and robust physical structure. Hollow species tend to grow faster than hummock species, resulting in generally higher net primary productivity in hollows than on hummocks [14], [19], [20]. In addition, *Sphagnum* species lack roots or stomata [21], meaning that their water status, survival, and dispersal are more tightly regulated by the climate compared to vascular plants, which can regulate transpiration and carbon uptake through their roots and stomata.

Considering the departure of the current climate from that of the early to mid-Holocene, when most peatlands were formed [22–24], it is not clear how climate change might affect *Sphagnum* species (the dominant peat-forming group) and *Sphagnum* peatland distributions in general. Some studies suggest that warming could enhance carbon sequestration by increasing *Sphagnum* net primary productivity [25], [26]. However, other findings indicate that carbon sequestration could decrease [27] because warming is expected to relax constraints on microbial decomposition, which would cause a decline in the carbon storage in northern peatlands [28–30].

The lack of congruence between these findings can be explored by understanding the important environmental factors influencing *Sphagnum* distributions and how changes in these factors might affect the distribution of *Sphagnum* peatland. Peatlands are formed by two major processes: paludification (from poor drainage) and terrestrialization (infilling of a water body by organic and inorganic sources), with paludification being more common [31]. While paludification is often initiated by stochastic events such as excessive precipitation, peatland formation through paludification likely occurs in regions where the prevailing climate can sustain the supply of water [31], [32]. That is, for peatland development to occur, the prevailing conditions must favour *Sphagnum* growth and dispersal. Thus, the distribution of *Sphagnum* species can be used as a proxy for understanding the environmental correlates of peatland distributions. This can be accomplished using bioclimatic species distribution models that integrate a species’ spatial occurrences with co-occurring environmental variables (e.g., temperature, precipitation) to model the species’ current distribution [33]. The relevant environmental variables can then be combined with future climate projections to project the species’ future climate distribution.

Although variants of bioclimatic models have been used previously to describe *Sphagnum* or *Sphagnum*-peatland distributions, those models were generated at a low resolution (~50 x 50 km) using coarse environmental variables such as mean annual temperature (MAT) and mean annual precipitation (MAP) [34], [35]. MAT and MAP can be poorly discriminating in terms of matching the spatial characteristics (or physiology) of species to their environments [36], [37]. For instance, Gunnarson [38] found that global productivity of *Sphagnum* increases with increasing MAT and MAP, but this is true for many species found in northern climates and is not necessarily informative for effectively linking *Sphagnum* species growth with their patterns of distribution. Although peatland typically occurs within a MAT of -12 to 5°C and a MAP of 200–1000 mm (which falls within the boreal axis), there is evidence that peatlands occupy a much broader and potentially drier climate extent [39]. Furthermore, MAT and MAP are often correlated with several other variables, and when incorporated into a model, could mask the effects of more physiologically important variables. Climate seasonality and local factors such as topography and geology also influence peatland formation and persistence [39–41].

Here, we use a simple but novel data integration method to assess the environmental attributes and potential distribution of *Sphagnum* peatland under current and future climates. Specifically, we use *Sphagnum* species distributions as a proxy for predicting *Sphagnum* peatland distribution and ask how species coexistence and climate variables influence the spatial pattern of *Sphagnum* peatland distribution under current and projected climate conditions. We address these questions using single- and multi-species (integrated records) distribution models generated at 1-km resolution to evaluate the environmental attributes of *Sphagnum* species and *Sphagnum*-peatland distributions in North America. We then use these correlates to assess how climate change might affect the future distribution of peatlands in North America.

## Methods

### Species occurrence data sets

We extracted a total of 2566 occurrence (longitude and latitude) records for four common *Sphagnum* species found throughout North America from Duke University herbarium (http://herbarium.duke.edu/databases), Consortium of North American Bryophyte Herbaria (www.bryophyteportal.org), and Consortium of Pacific Northwest Herbaria (www.pnwherbaria.org) databases. To reduce the influence of potentially uneven species detection efforts across the study region, a maximum of one record per species was used per 1-km^2^ grid cell. This resulted in a total of 1496 occurrence records that were used in modelling, comprising 289 *Sphagnum angustifolium*, 435 *S*. *fuscum*, 414 *S*. *magellanicum*, and 358 *S*. *rubellum* records. Although *Sphagnum* species can be difficult to identify, some such as *S*. *fuscum* can be identified with relative ease. Although misidentifications may influence the modelled distribution of an individual species, we integrated occurrence records for some models, so we expect such occurrence errors to have negligible effects for determining peatland distributions. Also, because of their lack of roots and stomata, *Sphagnum* species are opportunists and rarely occur individually, facilitating adequate representation of peatland habitat. Finally, integrating records from multiple databases that assemble records from expert sources should minimize errors due to misidentification and spatial bias.

**Fig 1:**
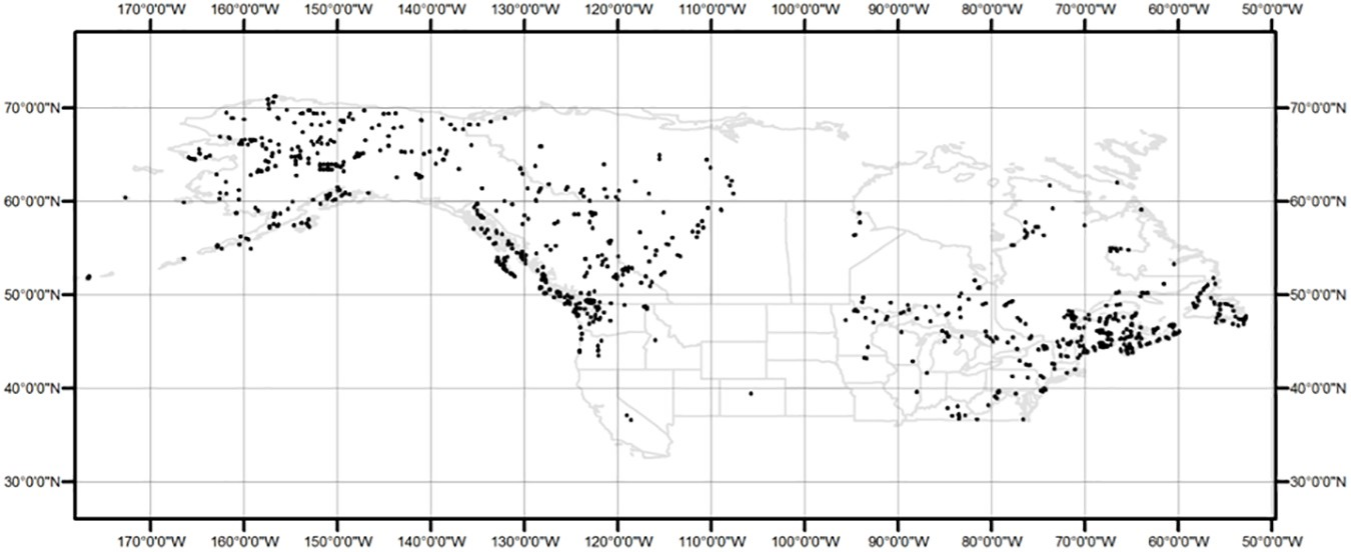
Map of the study area showing the distribution of all *Sphagnum* records used in this study.

For comparison with individual species distributions, we generated two data sets of multi-species occurrence records: “peatland” and “all species”. Well-developed *Sphagnum* peatlands are characterized by hummock-hollow microtopography, and *S*. *fuscum* is typically found on hummocks. Thus, if *S*. *fuscum* occurs (i.e., shares the same coordinates) with one or more other typical *Sphagnum* species, there is a high probability that *Sphagnum* peatland occurs in that location and a low probability that the records were collected in non-peatland habitat. The peatland data set comprised all records where *S*. *fuscum* coincided with at least one of the other three species (34, 104 and 72 records of *S*. *angustifolium*, *S*. *magellanicum* and *S*. *rubellum*, respectively; total of 210 occurrences). We used *S*. *fuscum* in this respect because it is one of the most common and abundant peat-forming *Sphagnum* species in northern peatlands (see [42]) and it is also easier to identify than most *Sphagnum* species. In contrast, the all-species data set comprised all unique records from all four *Sphagnum* species. The spatial distribution of these records across North America (Fig 1 see also S1 Fig) is consistent with the *Sphagnum* peatland distribution inferred from spore records [24].

The models were limited to the spatial extent of North America, north of the 37th parallel for the following reasons. Although some *Sphagnum* species have circumboreal or cosmopolitan distributions (e.g., *S*. *magellanicum* also occurs in South America), peatland does not always occur where there is *Sphagnum*. Furthermore, the greatest individual abundances should occur within the optimal range of environmental tolerances, and *Sphagnum* species are mainly northern plants. Finally, the large latitudinal gradient and high climate differential covered by records from North America should be sufficient to include the climate envelope of most *Sphagnum* species.

### Environmental data

We extracted altitude and 19 bioclimatic variables related to temperature and precipitation from WorldClim [43] for the current (1950–2000) climate and three general circulation models (GCMs) for the 2050 climate scenario based on representative concentration pathway +2.6. We used three GCMs because there is greater variability among GCMs than scenarios [44]. The GCMs were the Hadley Centre Global Environmental Model (HadGEM2-ES), the NASA Goddard Institute for Space Studies model (GISS-E2-R), and the Beijing Climate Center Climate System Model (BCC-CSM1-1). We also derived two additional variables for current and future climate: growing season temperature (GST) and soil moisture deficit (SMD). GST was calculated as the mean of May–August temperature; SMD was calculated as the difference between annual precipitation and potential evapotranspiration (PET) such that higher values of SMD indicate wetter areas and lower values indicate drier areas. PET data were obtained from the Consortium for Spatial Information (www.cgiar-csi.org; [45]). In total, we screened 22 variables as potential predictors by calculating Pearson correlation coefficients within the environmental space of the study area in ArcGIS 10.1 (ESRI, Redlands, CA; S1 Table). After screening, five biologically relevant variables that had little correlation across the environmental space were retained for modelling: temperature of the driest quarter (TDQ), temperature of the wettest quarter (TWQ), precipitation of the warmest quarter (PWQ), GST, and SMD. All environmental variables were extracted at 30 arc seconds (~1 km^2^) resolution. The study area covers most of North America but excludes areas south of the 37th parallel (~36°30’ N in eastern North America), with the exception of Nevada and California (Fig 1).

### Model procedure and evaluation

We first generated models using Maxent 3.3.3k [46], which is a presence-only modelling algorithm that incorporates the spatial attributes of a species with the environmental conditions that constrain its distribution to predict areas of suitability for that species [47]. Maxent models the distribution of a species as a logistic function of the environmental variables. Details of this method are discussed extensively elsewhere [47–50]. We generated 18 current models (3 GCMs x 6 data sets) and projected these models to 2050 for four single-species, one peatland, and one all-species data sets. We generated 10 replicates for each model using cross-validation by splitting the records into k = 10 folds (equal size subsamples), with k– 1 folds used to train the models and the remaining 1 fold used to test the models. Cross-validation limits spatial bias and model over-fitting. To allow model comparisons across species, we used the hinge feature, which generates easily interpreted non-linear models similar to generalized additive models [49]. For comparison, we generated additional models using the “all features” default function, which allows fitting of combinations of different statistical features for the models. The results were similar and are not reported here. We also explored the effects of MAT and MAP on model performance by including these variables as additional predictors in the models for peatland and all-species records. We used a 10th percentile training presence threshold such that suitable locations excluded the 10% least suitable occurrence locations [48]. This threshold was selected because some occurrence records may occur outside peatlands, and the threshold excludes locations that may be atypical for the species.

Models were evaluated using the area under the receiver operating characteristic curve (AUC), where a score of 0.5 is random, between 0.8 and 0.9 is good, and > 0.9 is excellent [51]. The importance of each environmental variable was evaluated using three indices: percent contribution of individual variables in explaining the variance in the model; a jackknife test, which indicates the model’s performance in the absence of a variable compared to that in its presence; and permutation importance, which estimates the decline in the model AUC as the variables are permutated randomly across the data set.

We investigated the disparities in the extent of suitable area between each current model and its future projection based on estimates of niche breadth calculated using ENMTools [52]. Niche breadth ranges from 0–1 and represents the range of environments that a species can inhabit [53]; it allows quantitative estimates of changes in peatland distribution between current and future models. We estimated the extent of spatial deviation between models by calculating pair-wise niche overlap (ranges from 0–1) using ENMTools. We determined the extent of agreement among the three future projections for each species distribution model by computing and mapping their coefficients of variation.

Finally, we divided the suitability scores into three classes to enable qualitative and quantitative assessments of the pattern of suitability between the models. We classified the model results such that a probability threshold of 0.2 = absence, 0.2–0.4 = low suitability, 0.4–0.6 = medium suitability, and 0.6–1 = high suitability locations. The 0.2 threshold represents the probability (to one decimal place) above which an environment is deemed suitable based on the 10th percentile training presence threshold that was applied to the models. We calculated the number of pixels in each suitability class > 0.2 and compared current models and future projections for the amount of area in each suitability class. For this comparison, we averaged across the three GCMs for each model.

To explore further the strength of pattern predicted for the *Sphagnum*-peatland distribution, we generated consensus models for current climate and each GCM for the all-species and peatland data sets. The consensus models were generated based on four modelling algorithms: flexible discriminant analysis [54], multivariate adaptive regression splines (MARS; [55]), random forest (RF; [56] and classification tree analysis (CTA; [57]. The models were implemented in the BIOMOD modelling platform [58] in R. Because these algorithms require presence-absence records, we randomly generated pseudo-absences that equalled half the size of each data set [59]. We implemented data splitting, where 70% of the records were used for training the models and 30% for testing, and each model was run twice. A model was included in the consensus model if it had AUC > 0.75 and true skill statistics (TSS) > 0.4; these are considered minimum evaluation scores for a useful model (e.g. [60]). Because variable importance for this modelling approach is not standardized across model algorithms, and given that there is no option for applying a standardized threshold, the consensus models were not quantitatively compared with the Maxent models and were displayed as binary maps, where locations with values ranging from 0 to 0.5 were deemed unsuitable and locations with values > 0.5 were considered suitable.

## Results

Model AUC scores ranged from 0.79–0.86 (Table 1) for Maxent models, indicating generally good discrimination of presence locations from other locations [51]. The single-species models performed similarly to the peatland model, whereas the all-species model performed slightly less well than the other models (Table 1). Except for RF (AUC 0.9), all consensus model algorithms had comparable AUC with the Maxent models. However, only RF performed consistently well.

**Table 1:** Percentage contributions of variables that were retained in the final models and the AUCs of each model.

	Model					
Climate variable	*S*. *angustifolium*	*S*. *fuscum*	*S*. *magellanicum*	*S*. *rubellum*	Peatland	All species
**Soil moisture deficit, SMD**	30.4	49.0	33.3	63.1	50.7	46.3
**Temperature of driest quarter, TDQ**	49.8	34.0	41.9	26.9	35.6	41.8
**Growing season temperature, GST**	13.2	14.6	10.7	6.4	10.3	9.7
**Precipitation of warmest quarter, PWQ**	5.2	1.1	12.9	1.0	1.4	1.0
**Temperature of wettest quarter, TWQ**	1.5	1.2	1.1	2.5	1.8	1.3
**AUC**	0.82	0.82	0.85	0.83	0.86	0.79

**Fig 2:**
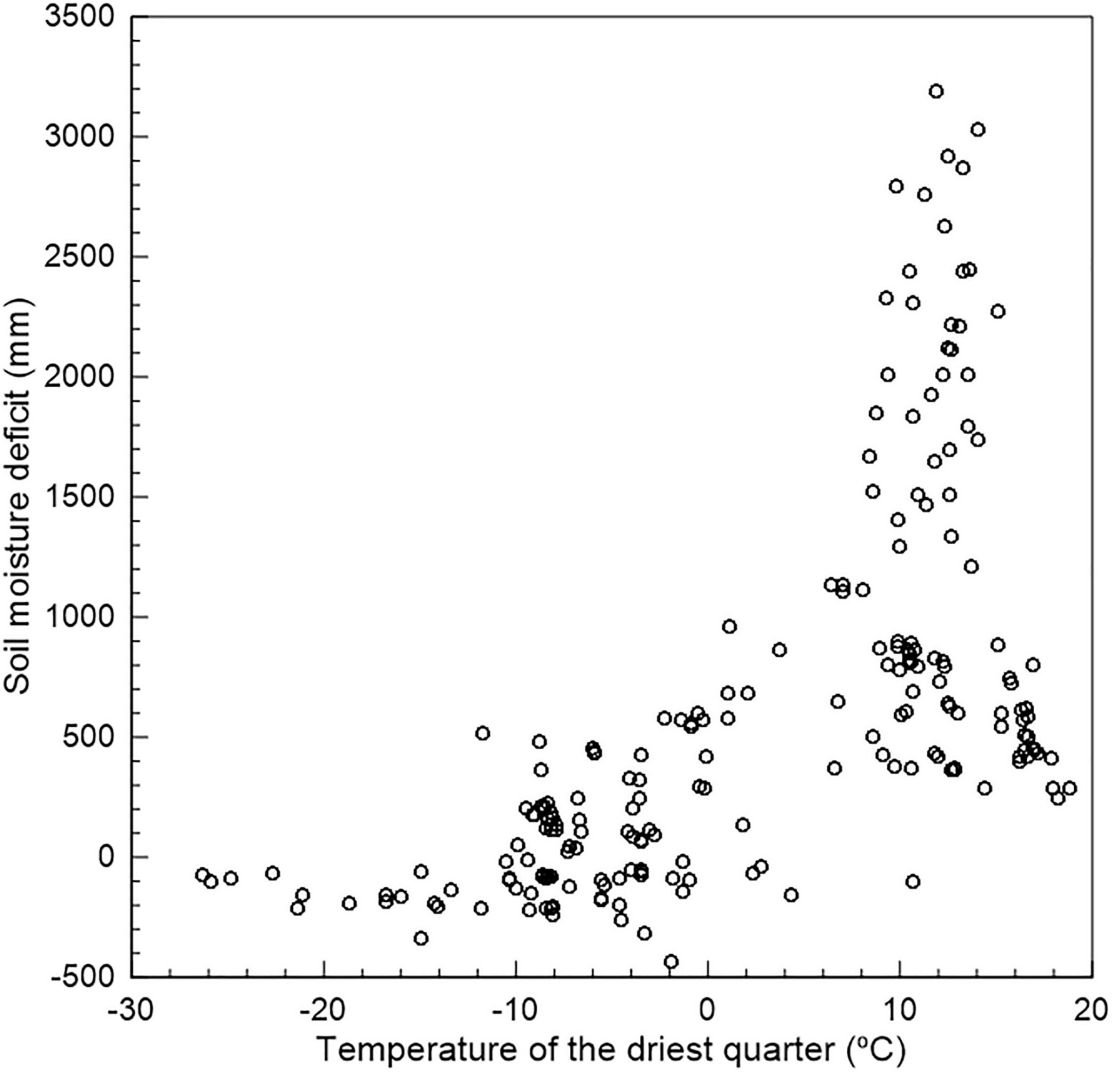
Relationship between soil moisture deficit (SMD) and temperature of the driest quarter for locations identified as peatlands. Peatlands were determined as co-occurrences of *Sphagnum fuscum* with one of *S*. *angustifolium*, *S*. *magellanicum* or *S*. *rubellum*. SMD is the difference between annual precipitation and potential evapotranpiration such that higher values indicate wetter areas.

The environmental variables SMD and TDQ explained the greatest amounts of model variance (30.4–63.1% and 26.9–49.8%, respectively; Table 1). In addition, SMD and TDQ were positively correlated (within the peatland data set: Spearman ρ = 0.71, N = 210, P < 0.0001), with peatlands having low SMD (drier) at temperatures below approximately 5°C and a greater range of SMD at temperatures > 5°C (Fig 2). GST explained moderate amounts of model variance (6.4–14.6%) and was uncorrelated with SMD (ρ = -0.05, N = 210, P = 0.5) and only weakly correlated with TDQ (ρ = 0.29, N = 210, P < 0.0001). PWQ and TWQ explained relatively little model variance (Table 1). Permutation importance and jackknife tests also indicated that SMD, TDQ and GST were the most important variables in the models (see S2 Table). This was also consistent for the consensus models. Given that TDQ ranged from -26.3 to 18.8 whereas MAT ranged from -13.9 to 11.3 for the peatland records, we further explored the peatland records within this climate space. For TDQ, most of the records < 0°C are located inland, whereas those > 1°C are more coastal. This pattern is not observed for MAT. As expected, both TDQ and MAT, and SMD and MAP, were correlated (ρ = 0.84, N = 210, P < 0.0001 and ρ = 0.95, N = 210, P < 0.0001, respectively). Furthermore, when MAP and MAT were included in the models, MAP masked the contribution of SMD, and the models were generally less interpretable because the model evaluation metrics contradicted one another (see S3 Table).

Niche breadths for the all-species distributions were 9.7–33.4% and 9.7–33.1% broader than those of the other current models and future projections, respectively (S4 Table). Compared to peatland, the all-species niche breadth was 33.4% and 30.1–33.1% broader for current and future models, respectively, and the niche overlap was 82% and 78–84% for current and future models, respectively. The niche overlap between the all-species and each of the single-species distributions was 83–93% for the current models and 77–93% for future projections; the niche overlap between the peatland and each of the single-species distributions was 80–88% for the current models and 77–89% for the future projections. The coefficients of variation for the future projections were generally low (< 0.1) in areas predicted as suitable, indicating good agreement among the projections (S2 Fig).

**Fig 3:**
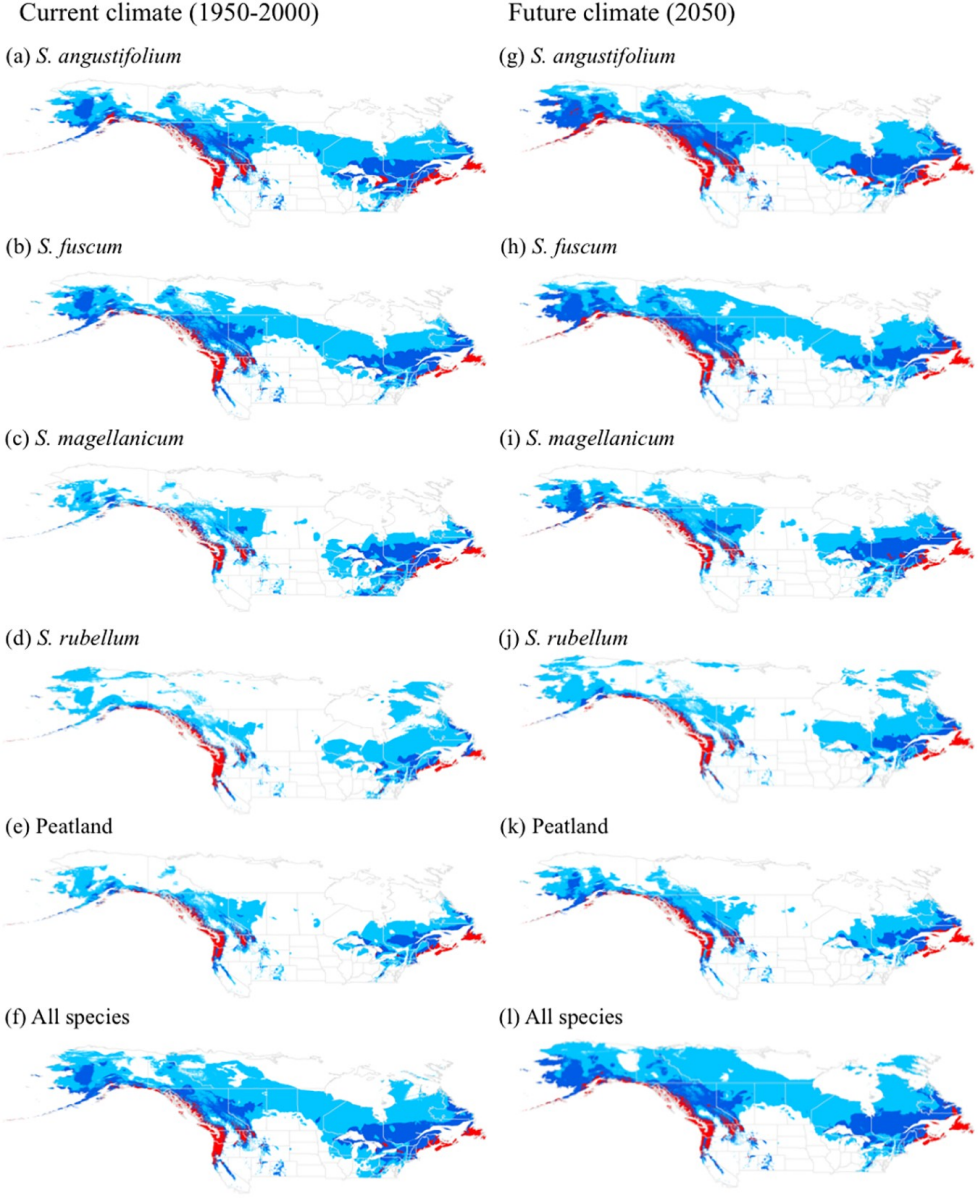
*Sphagnum* species distributions modelled under current and future climate projections using Maxent. Future maps indicate mean suitability based on three general circulation models. Red, highly suitable; blue, moderately suitable; turquoise, low suitability climate; white, unsuitable.

Although the four single-species models differed somewhat in extent and climate suitability, the core high-suitability areas and large-scale patterns of distribution were similar (Fig 3A–3D). The four single-species models and two multi-species models consistently showed that the suitability for *Sphagnum*-peatland distribution in North America is highest in coastal regions, particularly along the coasts of Washington, Oregon, and British Columbia in the west, and for most of Newfoundland and Nova Scotia and the coasts of New Brunswick and Maine in the east (Fig 3A–3F). Although there are some areas of high suitability in the continental interior, particularly in northern Idaho, southern Alberta, and southern Quebec, the suitability for *Sphagnum* peatland occurrence in the continental interior is generally low to moderate, especially in the north-central areas, which are generally cooler and drier.

**Fig 4:**
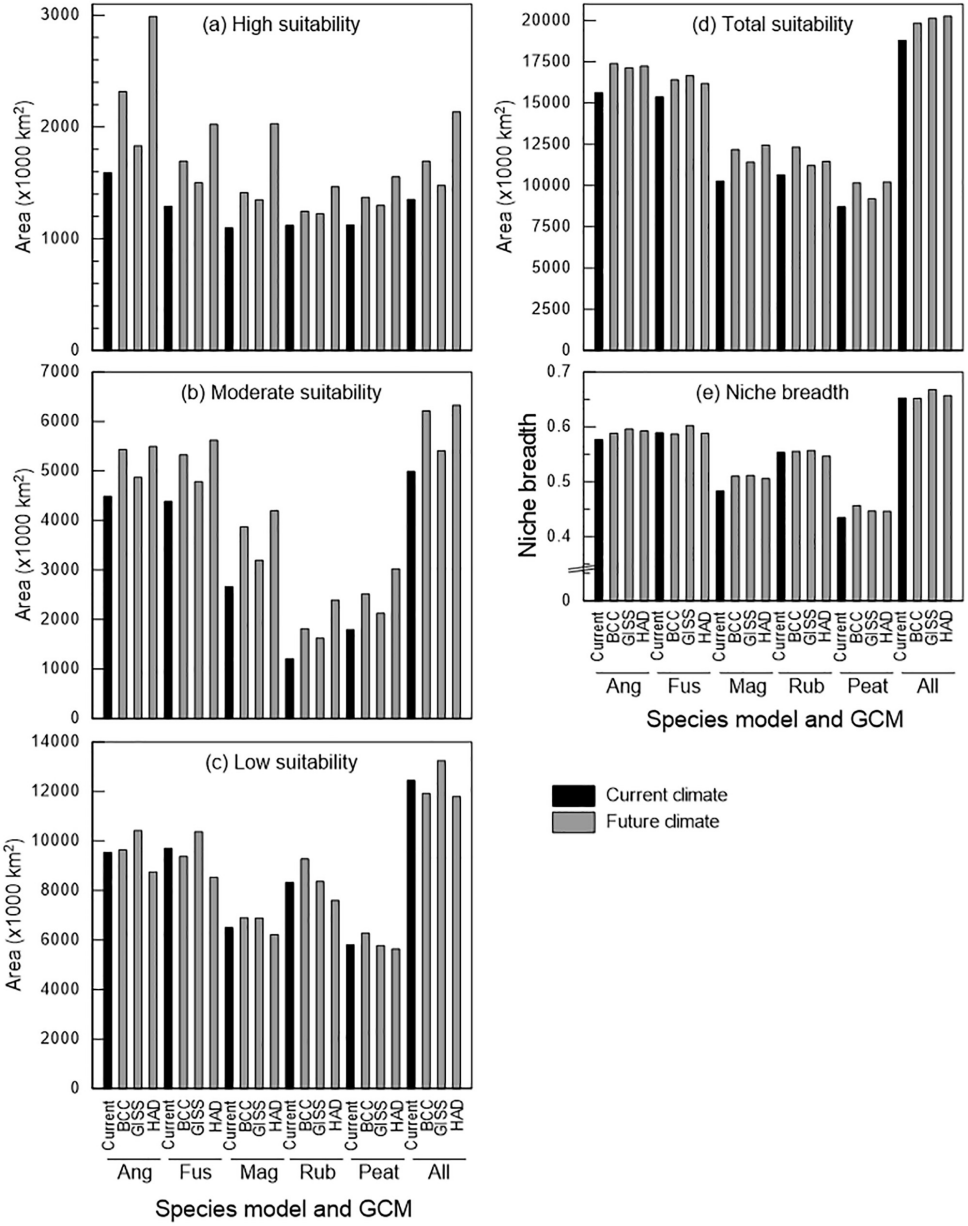
**Amount of area projected to be suitable currently and in 2050 under three general circulation models (GCMs; a-d), and niche breadth (e) for the six species models.** (a) High, (b) moderate, and (c) low suitability classes, and (d) total area deemed climatically suitable. Species-models: Ang, *Sphagnum angustifolium;* Fus, *S*. *fuscum;* Mag, *S*. *magellanicum;* Rub, *S*. *rubellum;* Peat, peatland; All, all species. GCMs: BCC, Beijing Climate Center Climate System Model (BCC-CSM1-1); GISS, NASA Goddard Institute for Space Studies model (GISS-E2-R); HAD, Hadley Centre Global Environmental Model (HadGEM2-ES). Note that the scale of the y-axis differs among panels.

**Fig 5:**
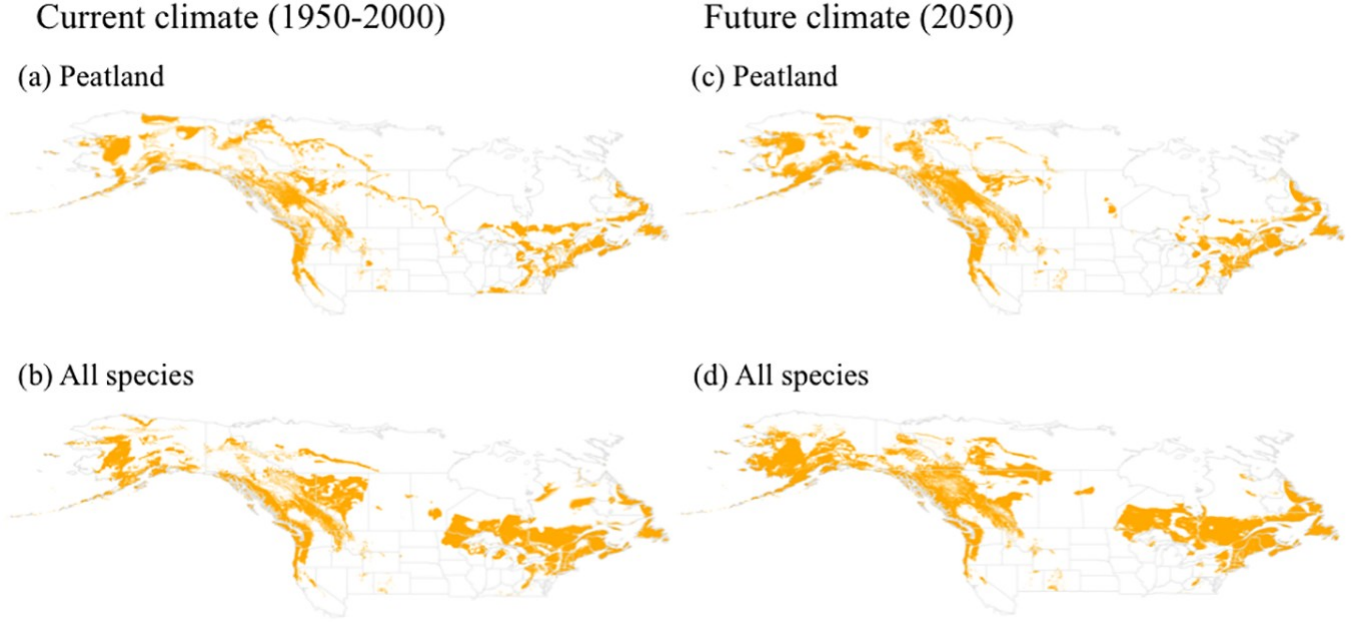
Consensus maps for *Sphagnum* species distributions modelled under current and future climate projections using four species distribution modelling algorithms. The four algorithms were flexible discriminant analysis, multivariate adaptive regression splines, random forest, and classification tree analysis. Future maps indicate mean suitability based on three general circulation models. Orange, suitable climate (suitability score > 0.5); white, unsuitable (suitability score < 0.5).

Total suitable area was greatest for the all-species model and lowest for the peatland model for both current and future climate; this difference was mainly driven by differences in the areas of low and moderate suitability (Fig 4). Although all three GCMs projected increases in total suitable area, there were differences among suitability classes, species models, and climate models (Table 2). Changes in low-suitability area were negative in half of the cases and were generally small, whereas increases in area were greatest for moderate suitability for the *S*. *rubellum* and peatland models, and greatest for high suitability for the remaining four models. The HadGEM2-ES climate model consistently projected greater areas of high suitability than did the other climate models across all species data sets (Table 2). For future projections, there were notable increases in high-suitability area on the coasts, but no to moderate increases in high-suitability area in the continental interior (Fig 3E and 3k). The pattern of suitability shown by Maxent for the core area of peatland distribution for current and future models is largely consistent with that of the consensus models (Fig 5).

**Table 2:** Percent change in suitable area compared to the current model for each species-climate model combination.

		Suitability class			
Species model	Climate model	Total	Low	Moderate	High
*S*. *angustifolium*	BCC-CSM1-1	11.3	1.0	21.0	45.6
	GISS-E2-R	9.6	9.2	8.5	15.1
	HadGEM2-ES	10.3	-8.3	22.3	87.8
*S*. *fuscum*	BCC-CSM1-1	6.7	-3.3	21.5	31.3
	GISS-E2-R	8.4	7.0	9.1	16.3
	HadGEM2-ES	5.2	-12.0	28.2	56.7
*S*. *magellanicum*	BCC-CSM1-1	18.7	6.1	45.3	28.7
	GISS-E2-R	11.4	5.9	20.0	22.7
	HadGEM2-ES	21.3	-4.3	57.5	85.0
*S*. *rubellum*	BCC-CSM1-1	15.8	11.3	50.7	11.2
	GISS-E2-R	5.2	0.5	34.7	9.2
	HadGEM2-ES	7.6	-8.7	98.9	30.9
Peatland	BCC-CSM1-1	16.6	8.1	40.5	22.2
	GISS-E2-R	5.5	-0.6	18.7	15.7
	HadGEM2-ES	17.2	-2.8	68.8	38.5
All species	BCC-CSM1-1	5.5	-4.2	24.5	25.4
	GISS-E2-R	7.2	6.4	8.4	9.4
	HadGEM2-ES	7.8	-5.2	26.8	58.2

## Discussion

Although we explicitly modelled the distribution of *Sphagnum* peatlands, the positive relationship between SMD and TDQ is consistent with the pattern of distribution of northern peatlands (see [61]). The lack of correlation between these variables in the whole environmental space but their correlation for peatland records means that they are biologically associated. However, unlike MAT, TDQ is a cross-seasonal variable and is able to capture the short-term climate distribution (dryness) that is more pertinent to the regional (coast versus inland) distributions of *Sphagnum*. The development and persistence of peatlands are controlled by the balance between precipitation and evaporation, and soil moisture availability in peatlands is determined by water availability or temperature [31]. In coastal regions, where precipitation occurs throughout the year, soil moisture is largely driven by precipitation; in continental regions, where the minimum summer temperature is relatively higher, temperature is the primary driver of soil moisture [62]. Thus, in many of the continental areas where *Sphagnum* peatlands occur (except those that are fed by perennial lakes), their persistence is likely maintained by the balance between low temperature and precipitation during dry periods, which is not adequately captured within MAP-MAT space. Whereas previous low-resolution models showed blanket distributions of *Sphagnum* peatland across North America (e.g., [34]), our models provide more fine-scale spatial discrimination. MAT and MAP lack discriminative power because climate variables are normally generated from daily temperature and precipitation, which are then processed into calendar months and further as annual variables. This multi-level processing means that annual variables tend to conceal the variability in the climate of many biological systems.

Choosing among single-species models and these two types of multi-species models would depend on their end use. Because our two types of multi-species models incorporate the environmental tolerances of multiple species in different ways, they may over- or underestimate the distribution of any one species. Thus, these types of models might not be well suited for conservation applications focusing on a single species. However, for species that have similar physiological requirements, such as *Sphagnum*, models generated from integrated records (i.e., co-occurrences) are likely more representative of a particular habitat distribution such as *Sphagnum* peatland. Therefore, this type of multi-species model is better suited to system-specific modelling. For instance, Oke et al., [63] used a system-specific query to retrieve occurrence records and model the distribution of nine plant species that typically co-exist in rock barrens, and the species were found to respond consistently to the same set of variables that characterizes rock barrens.

Although the models vary somewhat in their coverage, the general patterns of climate suitability are consistent, and the estimates of niche breadth, niche overlap, and suitable space consistently support the claim that climate warming could enhance *Sphagnum* productivity [26], [61], especially in coastal areas, which are the core areas of peatland distribution. This is consistent with the projection of increased precipitation for much of North America [64] and a positive response of plant productivity to warmer temperatures when moisture is not limiting [65]. Indeed, *Sphagnum* growth increases with warming and photoperiod [42], [66]. The models therefore suggest that warming would be effectively balanced by precipitation in places where *Sphagnum* peatlands typically occur. Because peatlands are believed to exist mainly under cool boreal climate, suitable space for *Sphagnum* peatlands might intuitively be expected to expand into the northern and north-central parts of North America with increased warming. However, these regions are characterized by cold, dry, arctic climate, and considering the stochastic nature of paludification, the prevailing conditions may not support frequent peatland initiation or *Sphagnum* growth [24], [35]. Furthermore, spore records indicate that the expansion of *Sphagnum* peatland distribution since the last glacial maximum (ca. 22–20 ka) has largely occurred in the eastern and western regions of North America, with limited north-central expansion [24]. Despite the projected increase in precipitation for the northern region, there is little indication that *Sphagnum* peatland would shift significantly northward.

Finally, because regional climates are strongly influenced by local geophysical factors such as large-scale orography (changes in air flow due to topographic interference), the pattern of *Sphagnum* peatland distribution projected for North America may not apply to *Sphagnum* peatlands in other regions, particularly those in Eurasia. For instance, Broccoli and Manabe [67] found that dryness in the continental interior of western North America is enhanced by the climate-modulating effect of the Rocky Mountains. Because excessive moisture is a prerequisite for *Sphagnum* colonization and peatland establishment, such large-scale topographic influences on regional climate distributions would likely play a vital role in the frequency of establishment and distribution of peatlands.

In conclusion, all models indicate that there could be a future increase in suitable climate area for *Sphagnum* peatlands, particularly in coastal regions. That is, at least in the immediate future, warming could be balanced by the projected increase in precipitation, which could enhance *Sphagnum* productivity. Future experimental studies that investigate the response of peatlands to warming and moisture levels should consider future climate projections in the design of such studies, especially those that manipulate peatland hydrology (i.e., water table drawdown). Although warming is occurring, increased precipitation is also projected for much of North America [64]. Uncritically drawing down the peatland water table or not balancing a drawdown with the corresponding temperature projections could result in misleading inferences concerning the link between temperature and moisture effects on peatland vegetation.

## Supporting information

S1 Table Correlation matrix for environmental variables screened for *Sphagnum*-peatland models. (DOCX)

S2 Table Permutation importance and jackknife results for *Sphagnum*-peatland models. (DOCX)

S3 Table Percent contributions, permutation importance (in parentheses) and jackknife tests for all-species and peatland models (Maxent) when MAP and MAP were included in the models. (DOCX)

S4 Table Niche breadth and niche overlap results for current and future *Sphagnum*-peatland distributions. (DOCX)

S1 Fig Maps of the study area, showing the spatial distributions of the records for each *Sphagnum*. (DOCX)

S2 Fig Coefficients of variation among the three future (2050) climate model projections for each species model. (DOCX)
