# Supplementary material for: Assessing environmental attributes and effects of climate change on Sphagnum peatland distributions in North America using single- and multi-species models
Source: PLoS One. 2017 Apr 20;12(4):e0175978. doi: 10.1371/journal.pone.0175978 (PMC5398565; doi:10.1371/journal.pone.0175978)
Supplement: S1 Table — (DOCX) [file pone.0175978.s001.docx]

**S1 Table.** Correlation matrix for environmental variables screened for *Sphagnum*-peatland models. Pearson correlation coefficients; highlighted cells indicate strong correlations (r > 0.7).

|  | **Bio1** | **Bio2** | **Bio3** | **Bio4** | **Bio5** | **Bio6** | **Bio7** | **Bio8** | **Bio9** | **Bio10** | **Bio11** | **Bio12** | **Bio13** | **Bio14** | **Bio15** | **Bio16** | **Bio17** | **Bio18** | **Bio19** | **Alt** | **GST** | **SMD** |
| --- | --- | --- | --- | --- | --- | --- | --- | --- | --- | --- | --- | --- | --- | --- | --- | --- | --- | --- | --- | --- | --- | --- |
| **Bio1** | 1.00 |  |  |  |  |  |  |  |  |  |  |  |  |  |  |  |  |  |  |  |  |  |
| **Bio2** | 0.66 | 1.00 |  |  |  |  |  |  |  |  |  |  |  |  |  |  |  |  |  |  |  |  |
| **Bio3** | 0.83 | 0.77 | 1.00 |  |  |  |  |  |  |  |  |  |  |  |  |  |  |  |  |  |  |  |
| **Bio4** | -0.73 | -0.33 | -0.82 | 1.00 |  |  |  |  |  |  |  |  |  |  |  |  |  |  |  |  |  |  |
| **Bio5** | 0.89 | 0.83 | 0.71 | -0.39 | 1.00 |  |  |  |  |  |  |  |  |  |  |  |  |  |  |  |  |  |
| **Bio6** | 0.93 | 0.49 | 0.87 | -0.91 | 0.69 | 1.00 |  |  |  |  |  |  |  |  |  |  |  |  |  |  |  |  |
| **Bio7** | -0.52 | 0.03 | -0.58 | 0.92 | -0.09 | -0.79 | 1.00 |  |  |  |  |  |  |  |  |  |  |  |  |  |  |  |
| **Bio8** | 0.38 | 0.34 | 0.05 | 0.17 | 0.54 | 0.11 | 0.31 | 1.00 |  |  |  |  |  |  |  |  |  |  |  |  |  |  |
| **Bio9** | 0.83 | 0.55 | 0.86 | -0.84 | 0.64 | 0.89 | -0.68 | -0.03 | 1.00 |  |  |  |  |  |  |  |  |  |  |  |  |  |
| **Bio10** | 0.91 | 0.70 | 0.64 | -0.39 | 0.98 | 0.71 | -0.14 | 0.61 | 0.63 | 1.00 |  |  |  |  |  |  |  |  |  |  |  |  |
| **Bio11** | 0.96 | 0.58 | 0.89 | -0.89 | 0.76 | 0.99 | -0.72 | 0.18 | 0.89 | 0.77 | 1.00 |  |  |  |  |  |  |  |  |  |  |  |
| **Bio12** | 0.49 | -0.05 | 0.34 | -0.58 | 0.20 | 0.56 | -0.61 | 0.02 | 0.48 | 0.29 | 0.54 | 1.00 |  |  |  |  |  |  |  |  |  |  |
| **Bio13** | 0.41 | -0.07 | 0.32 | -0.53 | 0.14 | 0.50 | -0.57 | 0.02 | 0.42 | 0.22 | 0.47 | 0.93 | 1.00 |  |  |  |  |  |  |  |  |  |
| **Bio14** | 0.40 | -0.09 | 0.22 | -0.49 | 0.13 | 0.46 | -0.52 | -0.02 | 0.41 | 0.23 | 0.45 | 0.86 | 0.65 | 1.00 |  |  |  |  |  |  |  |  |
| **Bio15** | -0.38 | -0.10 | -0.25 | 0.40 | -0.22 | -0.38 | 0.34 | 0.03 | -0.38 | -0.24 | -0.39 | -0.51 | -0.20 | -0.75 | 1.00 |  |  |  |  |  |  |  |
| **Bio16** | 0.43 | -0.06 | 0.33 | -0.53 | 0.16 | 0.51 | -0.57 | 0.02 | 0.43 | 0.24 | 0.49 | 0.94 | 0.99 | 0.66 | -0.24 | 1.00 |  |  |  |  |  |  |
| **Bio17** | 0.43 | -0.07 | 0.25 | -0.52 | 0.15 | 0.49 | -0.55 | -0.01 | 0.43 | 0.24 | 0.47 | 0.88 | 0.68 | 0.99 | -0.75 | 0.70 | 1.00 |  |  |  |  |  |
| **Bio18** | 0.35 | -0.06 | 0.03 | -0.22 | 0.17 | 0.27 | -0.22 | 0.39 | 0.14 | 0.30 | 0.29 | 0.74 | 0.63 | 0.75 | -0.47 | 0.64 | 0.76 | 1.00 |  |  |  |  |
| **Bio19** | 0.42 | -0.04 | 0.44 | -0.64 | 0.12 | 0.57 | -0.68 | -0.24 | 0.56 | 0.17 | 0.53 | 0.89 | 0.88 | 0.69 | -0.37 | 0.90 | 0.72 | 0.39 | 1.00 |  |  |  |
| **Alt** | 0.18 | 0.54 | 0.56 | -0.41 | 0.16 | 0.26 | -0.22 | -0.25 | 0.36 | 0.01 | 0.29 | -0.09 | -0.09 | -0.08 | -0.14 | -0.09 | -0.06 | -0.21 | 0.04 | 1.00 |  |  |
| **GST** | 0.93 | 0.71 | 0.67 | -0.43 | 0.97 | 0.74 | -0.18 | 0.60 | 0.66 | 1.00 | 0.79 | 0.32 | 0.26 | 0.25 | -0.26 | 0.27 | 0.27 | 0.32 | 0.20 | 0.04 | 1.00 |  |
| **SMD** | -0.25 | -0.66 | -0.32 | -0.09 | -0.53 | -0.10 | -0.32 | -0.30 | -0.13 | -0.42 | -0.15 | 0.70 | 0.69 | 0.63 | -0.26 | 0.69 | 0.64 | 0.55 | 0.64 | -0.28 | -0.40 | 1.00 |

**Variable descriptions**

BIO1 = Annual Mean Temperature

BIO2 = Mean Diurnal Range (Mean of monthly (max temp - min temp))

BIO3 = Isothermality (BIO2/BIO7) (* 100)

BIO4 = Temperature Seasonality (standard deviation *100)

BIO5 = Max Temperature of Warmest Month

BIO6 = Min Temperature of Coldest Month

BIO7 = Temperature Annual Range (BIO5-BIO6)

BIO8 = Mean Temperature of Wettest Quarter

BIO9 = Mean Temperature of Driest Quarter

BIO10 = Mean Temperature of Warmest Quarter

BIO11 = Mean Temperature of Coldest Quarter

BIO12 = Annual Precipitation

BIO13 = Precipitation of Wettest Month

BIO14 = Precipitation of Driest Month

BIO15 = Precipitation Seasonality (Coefficient of Variation)

BIO16 = Precipitation of Wettest Quarter

BIO17 = Precipitation of Driest Quarter

BIO18 = Precipitation of Warmest Quarter

BIO19 = Precipitation of Coldest Quarter

Alt = Altitude

GST = Growing season temperature (mean of temperature, May to August)

SMD = Soil moisture deficit (mean annual precipitation - potential evapotranspiration)
