# Supplementary material for: Assessing environmental attributes and effects of climate change on Sphagnum peatland distributions in North America using single- and multi-species models
Source: PLoS One. 2017 Apr 20;12(4):e0175978. doi: 10.1371/journal.pone.0175978 (PMC5398565; doi:10.1371/journal.pone.0175978)

**S2 Table.** Permutation importance and jackknife results for *Sphagnum*-peatland models. Results were obtained using Maxent software for four single-species and two multi-species distribution models.

**Permutation importance**

|  | Model | | | | | |
| --- | --- | --- | --- | --- | --- | --- |
| Climate variable | *S. angustifolium* | *S. fuscum* | *S. magellanicum* | *S. rubellum* | Peatland | All species |
| Soil moisture deficit, SMD | 38.8 | 32.7 | 41.4 | 33.3 | 29.5 | 33.5 |
| Temperature of driest quarter, TDQ | 30.3 | 42.5 | 25.5 | 41.6 | 51.2 | 48.8 |
| Growing season temperature, GST | 21.2 | 19.2 | 19.2 | 18.7 | 13.8 | 13.1 |
| Precipitation of warmest quarter, PWQ | 8 | 2.6 | 12.2 | 0.8 | 5 | 2.4 |
| Temperature of wettest quarter, TWQ | 1.8 | 3 | 1.8 | 5.5 | 0.5 | 2.2 |

**Jackknife results**

*Sphagnum angustifolium*


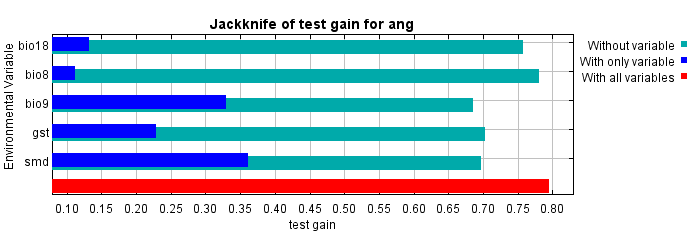


*Sphagnum fuscum*


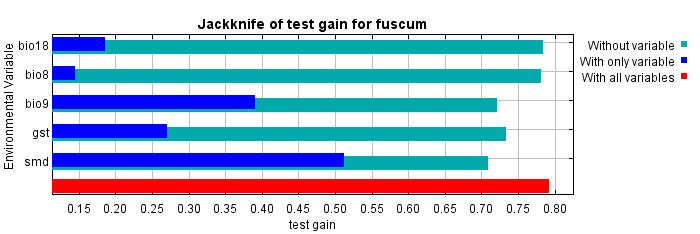


*Sphagnum magellanicum*


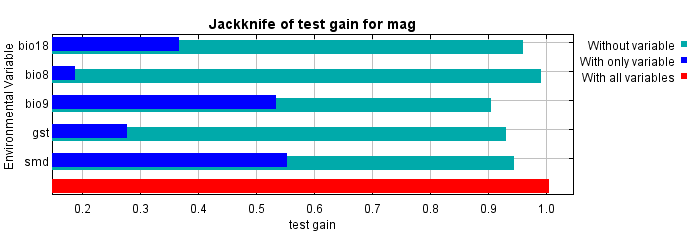


*Sphagnum rubellum*


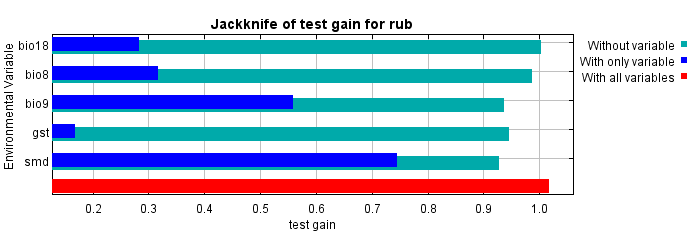


Peatland


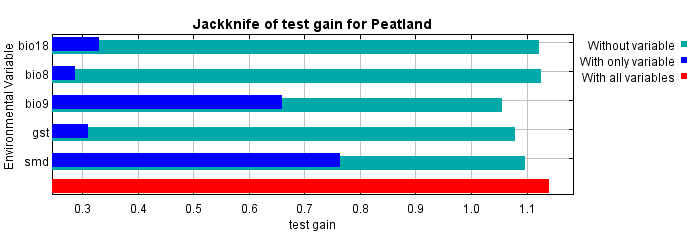


All species


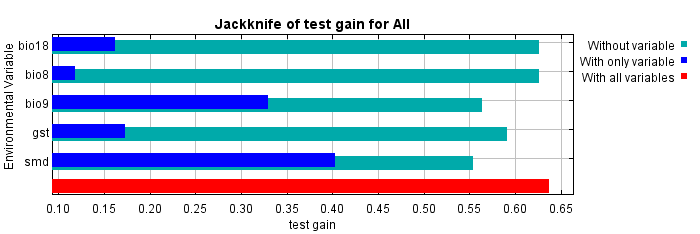

Supplement: S2 Table — (DOCX) [file pone.0175978.s002.docx]
