# Supplementary material for: Assessing environmental attributes and effects of climate change on Sphagnum peatland distributions in North America using single- and multi-species models
Source: PLoS One. 2017 Apr 20;12(4):e0175978. doi: 10.1371/journal.pone.0175978 (PMC5398565; doi:10.1371/journal.pone.0175978)

**S3 Table.** Percent contributions, permutation importance (in parentheses) and jackknife tests for all-species and peatland models (Maxent) when MAP and MAP were included in the models.

| Variable | All-species | Peatland |
| --- | --- | --- |
| MAP | 42.3 (13.1) | 35 (11.7) |
| TDQ | 22.1 (27.4) | 23.5 (31.6) |
| SMD | 17.3 (29.7) | 21.1 (22.2) |
| GST | 13.7 (11.1) | 14.7 (13.3) |
| PWQ | 2.6 (7) | 3.9 (11.6) |
| MAT | 1.6 (11.1) | 0.8 (9.3) |
| TWQ | 0.3 (0.5) | 1.1 (0.2) |


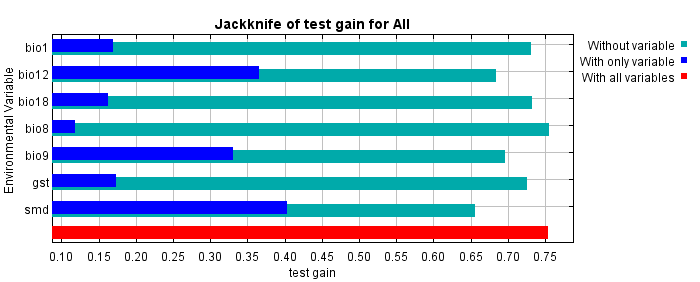

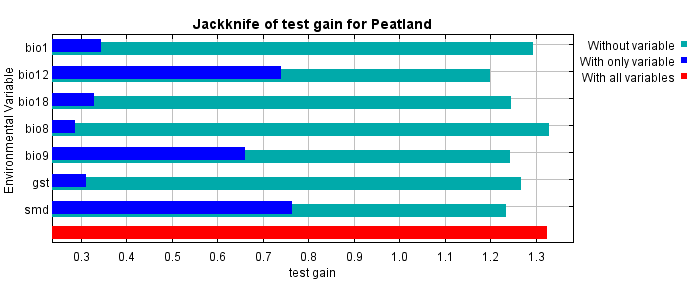

Supplement: S3 Table — (DOCX) [file pone.0175978.s003.docx]
