# Supplementary material for: Assessing environmental attributes and effects of climate change on Sphagnum peatland distributions in North America using single- and multi-species models
Source: PLoS One. 2017 Apr 20;12(4):e0175978. doi: 10.1371/journal.pone.0175978 (PMC5398565; doi:10.1371/journal.pone.0175978)
Supplement: S4 Table — (DOCX) [file pone.0175978.s004.docx]

**S4 Table.** Niche breadth and niche overlap results for current and future *Sphagnum*-peatland distributions. Results were obtained using Maxent software for four single-species and two multi-species distribution models, for current climate, and for future climate under three general circulation models (GCMs: BCC-CSM1-1, GISS-E2-R, and HadGEM2-ES).

**Niche breadth**

|  | Species model | | | | | |
| --- | --- | --- | --- | --- | --- | --- |
| Climate model | *S. angustifolium* | *S. fuscum* | *S. magellanicum* | *S. rubellum* | Peatland | All species |
| Current climate | 0.5767 | 0.5888 | 0.4829 | 0.5533 | 0.4347 | 0.6524 |
| BCC-CSM1-1 | 0.5883 | 0.5867 | 0.5101 | 0.5552 | 0.4564 | 0.6515 |
| GISS-E2-R | 0.5957 | 0.6019 | 0.5111 | 0.5567 | 0.4465 | 0.6677 |
| HadGEM2-ES | 0.5925 | 0.5878 | 0.5056 | 0.5467 | 0.4458 | 0.6568 |

**Niche overlap**

***Current climate***

| Species model | *S. angustifolium* | *S. fuscum* | *S. magellanicum* | *S. rubellum* | Peatland | All species |
| --- | --- | --- | --- | --- | --- | --- |
| *S. angustifolium* | 1 | 0.9226 | 0.8641 | 0.8328 | 0.8559 | 0.9223 |
| *S. fuscum* | 0.9226 | 1 | 0.8356 | 0.8411 | 0.8551 | 0.9274 |
| *S. magellanicum* | 0.8641 | 0.8356 | 1 | 0.7897 | 0.8825 | 0.8304 |
| *S. rubellum* | 0.8328 | 0.8411 | 0.7897 | 1 | 0.8016 | 0.8685 |
| Peatland | 0.8559 | 0.8551 | 0.8825 | 0.8016 | 1 | 0.8164 |
| All species | 0.9223 | 0.9274 | 0.8304 | 0.8685 | 0.8164 | 1 |

(See next page for future climate niche overlap)

**Niche overlap**

***Future climate***

| Species model |  | *S. angustifolium* | | | *S. fuscum* | | | *S. magellanicum* | | | *S. rubellum* | | | Peatland | | | All species | | |
| --- | --- | --- | --- | --- | --- | --- | --- | --- | --- | --- | --- | --- | --- | --- | --- | --- | --- | --- | --- |
|  | GCM | BCC | GISS | Had | BCC | GISS | Had | BCC | GISS | Had | BCC | GISS | Had | BCC | GISS | Had | BCC | GISS | Had |
| *S. angustifolium* | BCC | 1 | 0.9250 | 0.8959 | 0.9292 | 0.9016 | 0.8542 | 0.8785 | 0.8293 | 0.8471 | 0.8456 | 0.8237 | 0.8045 | 0.8704 | 0.8231 | 0.8119 | 0.9320 | 0.8938 | 0.8696 |
|  | GISS | 0.9250 | 1 | 0.8767 | 0.8793 | 0.9213 | 0.8300 | 0.8606 | 0.8751 | 0.8400 | 0.8343 | 0.8507 | 0.8019 | 0.8353 | 0.8598 | 0.7985 | 0.8953 | 0.9264 | 0.8539 |
|  | Had | 0.8959 | 0.8767 | 1 | 0.8997 | 0.8915 | 0.9216 | 0.8213 | 0.7927 | 0.8744 | 0.8477 | 0.8222 | 0.8591 | 0.8327 | 0.7992 | 0.8582 | 0.8962 | 0.8783 | 0.9293 |
| *S. fuscum* | BCC | 0.9292 | 0.8793 | 0.8997 | 1 | 0.9279 | 0.8967 | 0.8572 | 0.8064 | 0.8496 | 0.8515 | 0.8154 | 0.8277 | 0.8738 | 0.8152 | 0.8297 | 0.9302 | 0.8833 | 0.8858 |
|  | GISS | 0.9016 | 0.9213 | 0.8915 | 0.9279 | 1 | 0.8792 | 0.8440 | 0.8455 | 0.8450 | 0.8511 | 0.8534 | 0.8312 | 0.8448 | 0.8563 | 0.8174 | 0.9089 | 0.9280 | 0.8823 |
|  | Had | 0.8542 | 0.8300 | 0.9216 | 0.8967 | 0.8792 | 1 | 0.7893 | 0.7557 | 0.8493 | 0.8211 | 0.7903 | 0.8650 | 0.8168 | 0.7755 | 0.8619 | 0.8673 | 0.8426 | 0.9289 |
| *S. magellanicum* | BCC | 0.8785 | 0.8606 | 0.8213 | 0.8572 | 0.8440 | 0.7893 | 1 | 0.9047 | 0.8875 | 0.8139 | 0.7966 | 0.7797 | 0.8914 | 0.8642 | 0.8173 | 0.8532 | 0.8333 | 0.7948 |
|  | GISS | 0.8293 | 0.8751 | 0.7927 | 0.8064 | 0.8455 | 0.7557 | 0.9047 | 1 | 0.8572 | 0.7968 | 0.8200 | 0.7718 | 0.8315 | 0.8866 | 0.7861 | 0.8120 | 0.8464 | 0.7701 |
|  | Had | 0.8471 | 0.8400 | 0.8744 | 0.8496 | 0.8450 | 0.8493 | 0.8875 | 0.8572 | 1 | 0.8091 | 0.7905 | 0.8303 | 0.8601 | 0.8482 | 0.8906 | 0.8330 | 0.8200 | 0.8455 |
| *S. rubellum* | BCC | 0.8456 | 0.8343 | 0.8477 | 0.8515 | 0.8511 | 0.8211 | 0.8139 | 0.7968 | 0.8091 | 1 | 0.9215 | 0.8929 | 0.8272 | 0.8044 | 0.7959 | 0.8744 | 0.8613 | 0.8517 |
|  | GISS | 0.8237 | 0.8507 | 0.8222 | 0.8154 | 0.8534 | 0.7903 | 0.7966 | 0.8200 | 0.7905 | 0.9215 | 1 | 0.8695 | 0.7938 | 0.8193 | 0.7679 | 0.8497 | 0.8776 | 0.8266 |
|  | Had | 0.8045 | 0.8019 | 0.8591 | 0.8277 | 0.8312 | 0.8650 | 0.7797 | 0.7718 | 0.8303 | 0.8929 | 0.8695 | 1 | 0.8076 | 0.7913 | 0.8338 | 0.8303 | 0.8258 | 0.8830 |
| Peatland | BCC | 0.8704 | 0.8353 | 0.8327 | 0.8738 | 0.8448 | 0.8168 | 0.8914 | 0.8315 | 0.8601 | 0.8272 | 0.7938 | 0.8076 | 1 | 0.9006 | 0.8840 | 0.8363 | 0.8045 | 0.8006 |
|  | GISS | 0.8231 | 0.8598 | 0.7992 | 0.8152 | 0.8563 | 0.7755 | 0.8642 | 0.8866 | 0.8482 | 0.8044 | 0.8193 | 0.7913 | 0.9006 | 1 | 0.8502 | 0.7965 | 0.8203 | 0.7725 |
|  | Had | 0.8119 | 0.7985 | 0.8582 | 0.8297 | 0.8174 | 0.8619 | 0.8173 | 0.7861 | 0.8906 | 0.7959 | 0.7679 | 0.8338 | 0.8840 | 0.8502 | 1 | 0.7925 | 0.7750 | 0.8215 |
| All species | BCC | 0.9320 | 0.8953 | 0.8962 | 0.9302 | 0.9089 | 0.8673 | 0.8532 | 0.8120 | 0.8330 | 0.8744 | 0.8497 | 0.8303 | 0.8363 | 0.7965 | 0.7925 | 1 | 0.9347 | 0.9058 |
|  | GISS | 0.8938 | 0.9264 | 0.8783 | 0.8833 | 0.9280 | 0.8426 | 0.8333 | 0.8464 | 0.8200 | 0.8613 | 0.8776 | 0.8258 | 0.8045 | 0.8203 | 0.7750 | 0.9347 | 1 | 0.8871 |
|  | Had | 0.8696 | 0.8539 | 0.9293 | 0.8858 | 0.8823 | 0.9289 | 0.7948 | 0.7701 | 0.8455 | 0.8517 | 0.8266 | 0.8830 | 0.8006 | 0.7725 | 0.8215 | 0.9058 | 0.8871 | 1 |
