# Supplementary material for: Assessing environmental attributes and effects of climate change on Sphagnum peatland distributions in North America using single- and multi-species models
Source: PLoS One. 2017 Apr 20;12(4):e0175978. doi: 10.1371/journal.pone.0175978 (PMC5398565; doi:10.1371/journal.pone.0175978)

**S1 Fig.** Maps of the study area, showing the spatial distributions of the records for each *Sphagnum* species. Because of the scale, some points are obscured by overlapping.

*S. angustifolium*


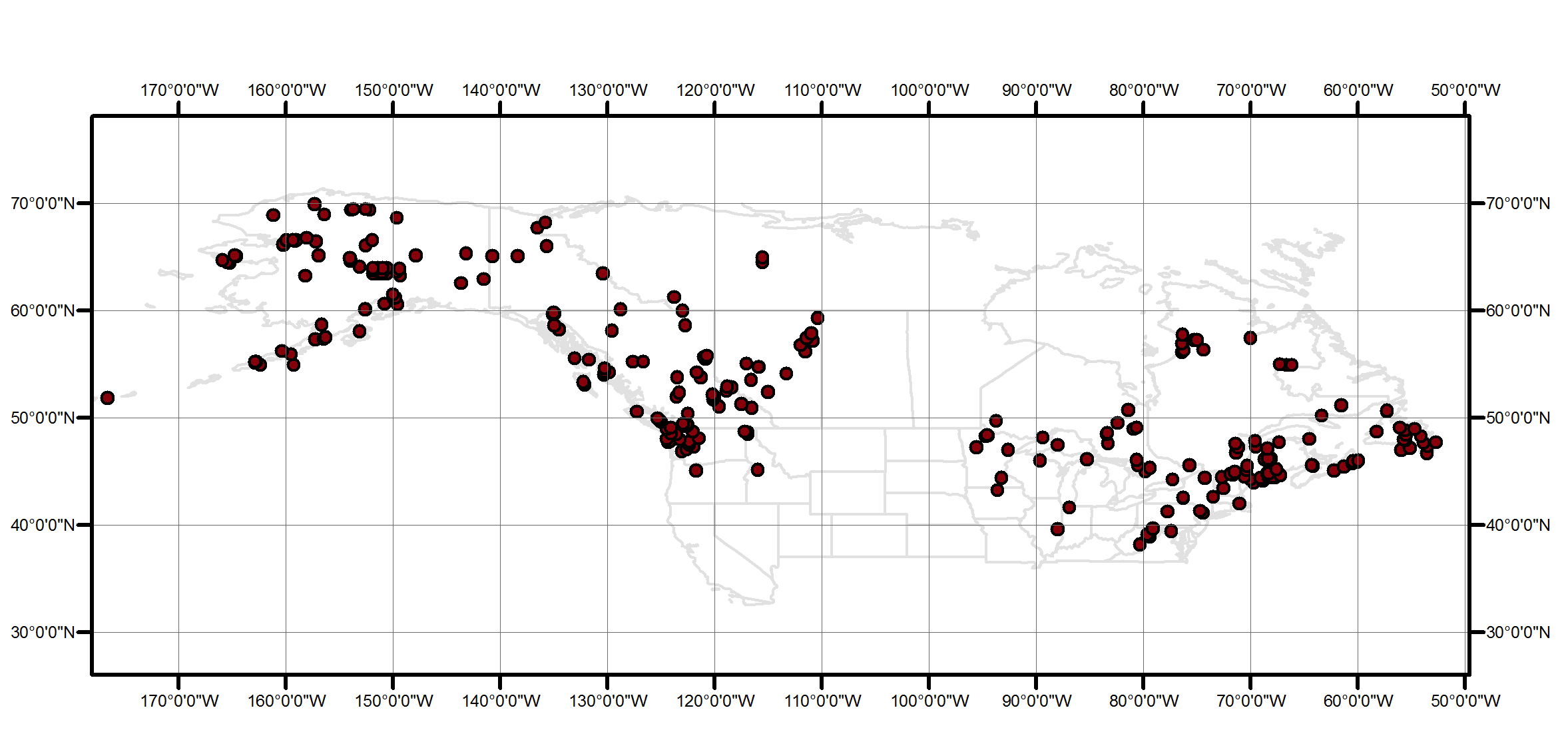


*S. magellanicum*


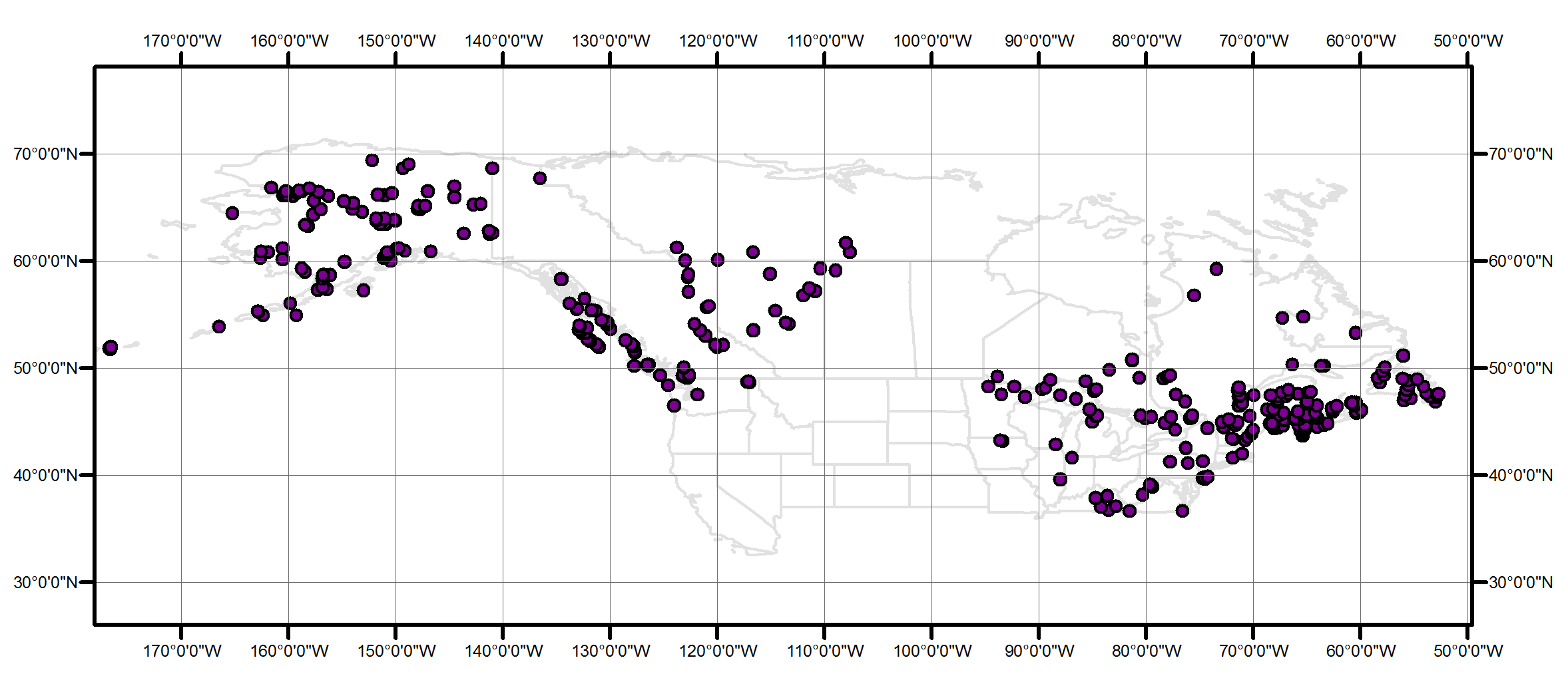


*S. fuscum*


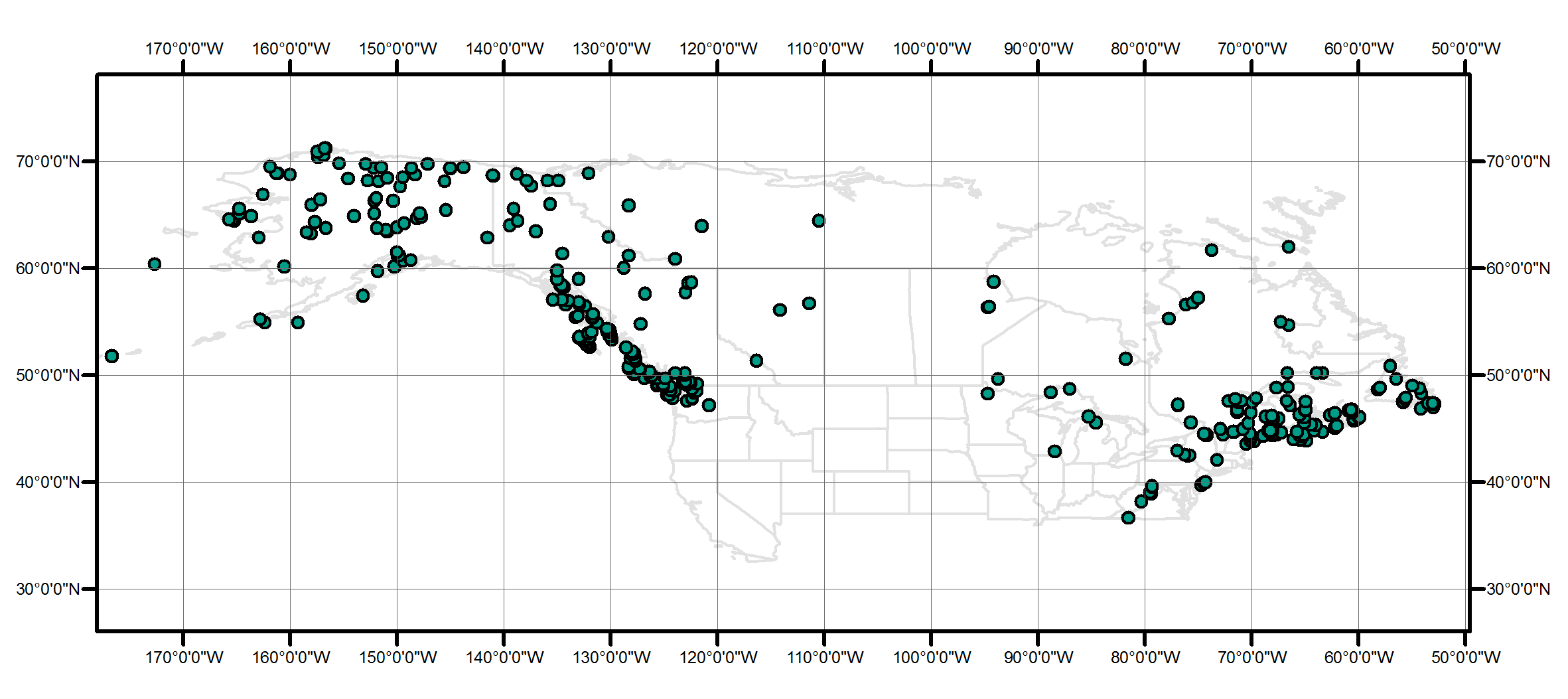


*S. rubellum*


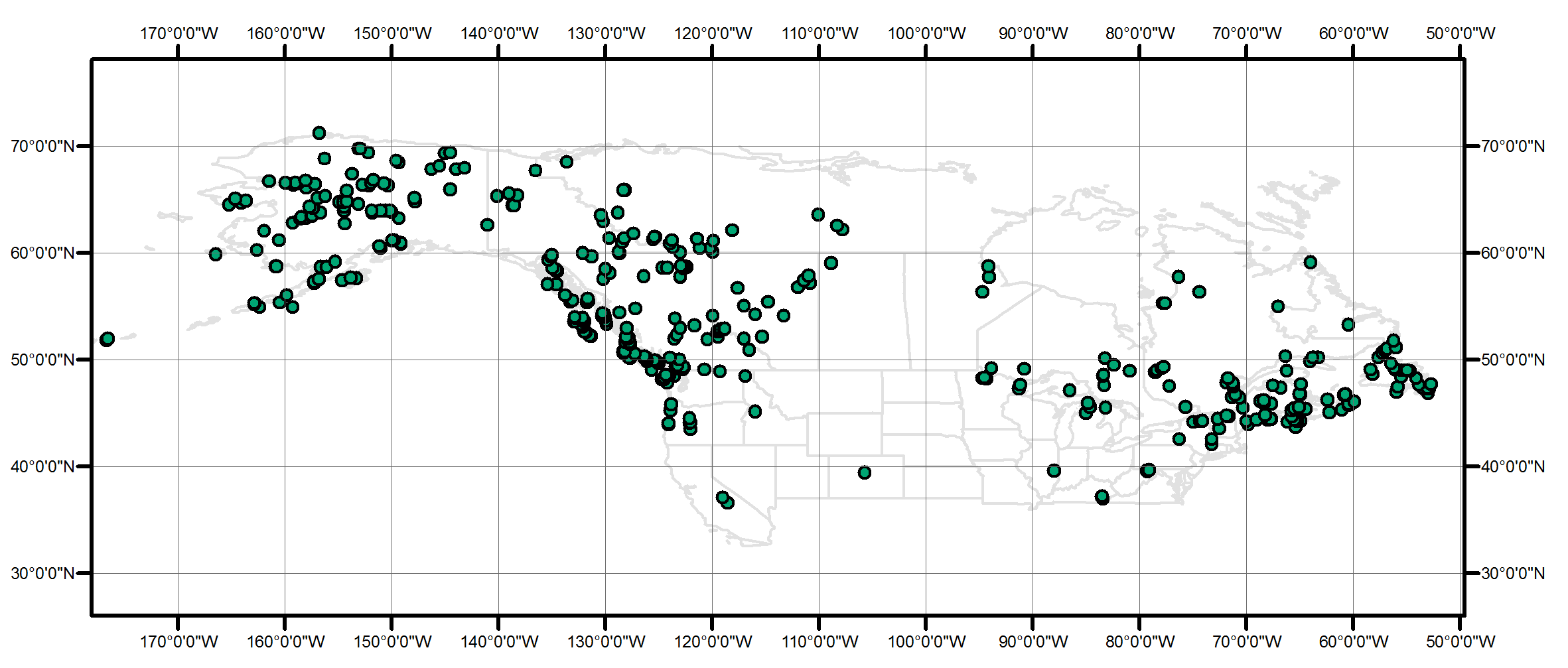

Supplement: S1 Fig — (DOCX) [file pone.0175978.s005.docx]
