# Supplementary material for: Assessing environmental attributes and effects of climate change on Sphagnum peatland distributions in North America using single- and multi-species models
Source: PLoS One. 2017 Apr 20;12(4):e0175978. doi: 10.1371/journal.pone.0175978 (PMC5398565; doi:10.1371/journal.pone.0175978)
Supplement: S2 Fig — (DOCX) [file pone.0175978.s006.docx]

**S2 Fig.** Coefficients of variation among the three future (2050) climate model projections for each species model.
